# Knowledge synthesis of benefits and adverse effects of measles vaccination: the Lasbela balance sheet

**DOI:** 10.1186/1472-698X-9-S1-S6

**Published:** 2009-10-14

**Authors:** Robert J Ledogar, John Fleming, Neil Andersson

**Affiliations:** 1 CIETinternational, 511 Avenue of the Americas #132, New York, NY, USA; 2 Centro de Investigación de Enfermedades Tropicales (CIET), Universidad Autónoma de Guerrero, Calle Pino, Acapulco, Mexico

## Abstract

**Background:**

In preparation for a cluster-randomized controlled trial of a community intervention to increase the demand for measles vaccination in Lasbela district of Pakistan, a balance sheet summarized published evidence on benefits and possible adverse effects of measles vaccination.

**Methods:**

The balance sheet listed: 1) major health conditions associated with measles; 2) the risk among the unvaccinated who contract measles; 3) the risk among the vaccinated; 4) the risk difference between vaccinated and unvaccinated; and 5) the likely net gain from vaccination for each condition.

**Results:**

Two models revealed very different projections of net gain from measles vaccine. A Lasbela-specific combination of low period prevalence of measles among the unvaccinated, medium vaccination coverage and low vaccine efficacy rate, as revealed by the baseline survey, resulted in less-than-expected gains attributable to vaccination. Modelled on estimates where the vaccine had greater efficacy, the gains from vaccination would be more substantial.

**Conclusion:**

Specific local conditions probably explain the low rates among the unvaccinated while the high vaccine failure rate is likely due to weaknesses in the vaccination delivery system. Community perception of these realities may have had some role in household decisions about whether to vaccinate, although the major discouraging factor was inadequate access. The balance sheet may be useful as a communication tool in other circumstances, applied to up-to-date local evidence.

## Background

In early 2005, preparations began for a cluster-randomized controlled trial of a knowledge translation (KT) intervention to increase the demand for measles vaccination in Lasbela district of Balochistan province, Pakistan. Separate articles discuss the protocol [1], baseline findings [2], and the outcome of the trial [3].

The team worked from a defined theoretical position. We assumed that household decisions are fundamentally rational, at least in the sense that families weigh up costs and benefits of having their children vaccinated [4-6].

Early in the planning stages for the trial, well before conducting and analyzing the baseline survey, we expected to encounter concern over the potential adverse effects of measles vaccine. Previous work on child vaccination in the region had encountered a number of negative views. There was also a small but important international literature on the subject. Three majority-Muslim States in Nigeria at one time or another stopped internationally sanctioned polio vaccination campaigns based on conspiracy theories that the vaccination was a Western plot to sterilize children and even spread HIV among them [7]. In Europe and North America concerns about possible connections between the MMR vaccine and autism had been circulating in the media for years [8].

If these or similar concerns were raised at community level in Lasbela, we anticipated they would have to be addressed with full and accurate information. We prepared for this KT by designing a tool for discussing with the communities specific gains and losses to be anticipated from measles vaccination - the Lasbela Vaccination Balance Sheet.

**Figure 1:**
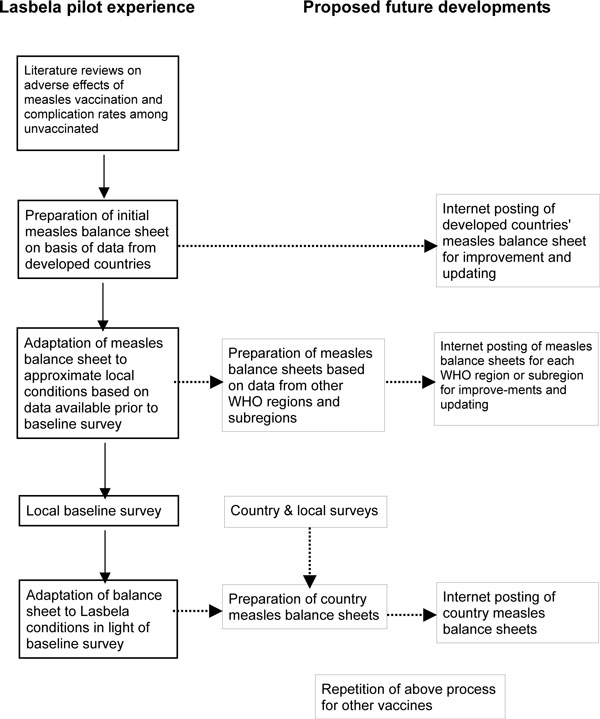
**Model of measles balance sheet development**.

The balance sheet went through a series of steps that are diagrammed in the left-hand column of Figure 1. Our first step was to gather the necessary data from developed-country sources from which almost all available estimates of measles complication rates and vaccination adverse effects are derived. After drawing up the balance sheet based on these estimates we then added parallel estimates for the major adverse effects under local conditions using whatever data were available on Lasbela, Balochistan, Pakistan or South Asia prior to our own baseline survey. The results of these steps are combined in Table 1. Outcomes of the baseline survey obliged us to modify our assumptions and recalculate the balance sheet. The main differences between our estimates prior to and after the baseline survey are presented in Table 2.

**Table 1:** Original Measles Vaccination Balance Sheet.

(1) Condition	(2) Measles complication rate among unvaccinated^a^	(3) Vaccine adverse event rate	(4) Risk Difference	(5) Number Needed to Treat (NNT)	(6) Expected Gain per thousand^e^
Diarrhoea in highly developed countries	0.08	0.0357	0.0443	23	30
**Diarrhoea - WHO South Asia subregion adjusted**	**0.41**	**0.0045**	**0.4055**	**3**	**277**
Bronchopneumonia in highly developed countries	0.01-0.06	0	0.01	100	7
**Bronchopneumonia - WHO South Asia subregion adjusted**	**0.089-0.53**	**0**	**0.089**	**11**	**61**
**Blindness (Africa)**	**0.04**^b^	**0**	**0.04**	**25**	**27**
Otitis media	0.07-0.09	0.0333	0.0367	27	25
Death in highly developed countries	0.001-0.003	0.0002	0.0008	1,189	0.6
**Death - WHO South Asia subregion data**	**0.0228**	**0.0002**	**0.0208**	**50**	**14**
Death in developing world (by some estimates)	15%	0.0002	0.1498	7	102
Convulsions	0.006-0.007^c^	0.0022	0.0038	266	3
Post-infectious encephalitis	0.001-0.0005	0.0004	0.0006	1,591	0.4
Subacute sclerosing panencephalitis (SSPE)	0.00001	0	0.00001	100,000	7/million
Anaphylaxis	0^d^	1-3.5 per million	-0.000001	-1,000,000	-7/ten million

^a ^Except where noted all figures in this column taken from Strebel et al [44]. ^b ^[45]. ^c ^[51]. ^d ^[33].

^e ^Gain = RDx(PRIxVER), where RD = risk difference, PRI = proportion requiring intervention (0.76), VER = vaccine efficacy rate (0.90).

The bold-font rows show the conditions for which the data are most applicable to the South Asia region or, at least, developing country conditions - diarrhoea, pneumonia, blindness and death.

**Table 2:** Anticipated population gains and losses - cases of the condition prevented or caused if all children currently unvaccinated were vaccinated^a^.

Condition	Expected gain (number prevented) under original assumptions	Expected gain (number prevented) applying efficacy rates from Lasbela baseline survey^b^
**Diarrhoea**	**277 per thousand**	**30 per thousand**
**Bronchopneumonia**	**61 per thousand**	**7 per thousand**
**Blindness**	**27 per thousand**	**3 per thousand**
Otitis media	25 per thousand	3 per thousand
**Death**	**14 per thousand**	**15 per ten thousand**
Convulsions	3 per thousand	3 per ten thousand
Post-infectious	4 per ten thousand	5 per hundred thousand
encephalitis	7 per million	7 per ten million
SSPE	Losses (original assumptions)	Losses after baseline survey
Anaphylaxis	7 per ten million	7 per hundred million

^a ^Sources for all calculations are the same as in Table 1. Differences in gains are due to revised assumptions following the baseline survey.

^b ^Gain = [RDx(PRIxVER)] × 0.361, where RD = Risk difference, PRI = proportion requiring intervention (0.49), and VER = Vaccine efficacy rate (0.415).

The bold-font rows show the conditions for which the data are most applicable to the South Asia region or, at least, developing country conditions - diarrhoea, pneumonia, blindness and death.

## Methods

A balance sheet synthesized published information on risks of adverse effects of vaccination and risks for the same outcomes as complications of naturally occurring measles. We presented the difference in risk of each outcome between vaccinated and unvaccinated children as the risk difference (RD). Where the literature provided a range for the unvaccinated, we used the lower figure in the calculation. The inverse of the RD is the number needed to treat (NNT), the number of persons that must be vaccinated in order to avoid one case of the negative outcome, and the number needed to harm (NNH), the number that must be vaccinated to produce one case of harm.

We presented the data in terms of "gains," the potential number of cases of the outcome (or complication) prevented if all children currently unvaccinated were vaccinated for measles. The gain is calculated by multiplying the RD by the proportion requiring the intervention (PRI) and adjusting for vaccine efficacy [9].

The first model of expected gains used our original assumptions about conditions in Lasbela, before analysis of the 2005 baseline survey. We applied a PRI of 0.76 based on the overall vaccination rate reported for Balochistan province in 2001-02 according to the Pakistan Integrated Household Survey, Round 4 [10]. We assumed a vaccine efficacy rate (VER) of 90%, which is at the low end of estimates provided in the literature for developed countries [11].

For adverse effects, we consulted a 2005 comprehensive review of measles, mumps and rubella (MMR) vaccine effects published by the Cochrane Collaboration [12]. Of 31 studies that met the Cochrane review's inclusion criteria, we selected those 20 that contained specific numbers of adverse effect cases together with the total study population [13-32]. We extracted these data to create tables for each adverse effect (AE table) in a spreadsheet workbook.

We linked the rate of occurrence for each adverse effect in the table to the appropriate cell in the summary balance sheet. Due to time and resource constraints we did not assign credibility weights to each study. A summary rate of occurrence is derived from the total of adverse event cases divided by the total of the study populations reported by our sources for each adverse effect. Where there were no population-based AE data to match with a known complication of naturally occurring measles we use rates estimated by Duclos and Ward [33].

To obtain complication rates among the unvaccinated with measles, we consulted World Health Organization (WHO) documents [34-36], a standard textbook on the subject [37] and the sources these cited. Most sources for complication rates among the unvaccinated provide figures based on conditions in the developed world, although some sources add broad estimates for rates of certain conditions in the developing world. In order to develop figures approximating conditions in Pakistan, we adjusted the complication rates for pneumonia and diarrhoea using regional incidence rates for these diseases (from all causes) based on data reported by WHO [38]. Pakistan is part of WHO's Eastern Mediterranean Region (EMRO), mortality substratum D. We calculated the rates of incidence of pneumonia (lower respiratory infections) and diarrhoea for EMRO-D and used European region substratum A as a comparison.

The adjustment for pneumonia applies the proportional difference in incidence rates for lower respiratory infections (from all causes) for the Euro-A and EMRO-D substrata (EMRO-D (42,687/351,256 = 0.12) Euro-A (5,649/412,512 = 0.01). The EMRO-D rate is 12 times the Euro-A rate (0.12/0.01). We used the same procedure for diarrhoea (EMRO-D (345,605/351,256 = 0.98) Euro-A (79,219/412,512 = 0.19). The EMRO-D incidence rate is 5.2 times that of Euro-A (0.98/0.19). The balance sheet reports figures for the base rate (developing world conditions) and EMRO-D adjusted rates (Pakistan conditions) for diarrhoea and pneumonia.

For a closer estimate of deaths due to naturally occurring measles in Pakistan, we divided the number of measles deaths for the year 2002 in WHO's EMRO-D region (70,392) [39] by measles incidence in that region (3,079,000) in the same year [40]. The rate thus derived is almost certainly underestimated. To provide an outer limit closer to reality in Pakistan, we also included gains calculations based on a death rate of 15%, which was suggested in other sources consulted as appropriate for developing countries [41]. It was assumed that virtually all the unvaccinated were likely to contract measles. In this we followed White and colleagues who reported that, according to United States records from the period previous to the introduction of measles vaccine, 95% of individuals in an unvaccinated population had evidence of measles infection by age 30 [42]. The vast majority of these infections are likely to have occurred in early childhood.

Blindness is usually reported as a complication of measles in the developing world, though not in the developed world. No base complication rates for the developing world were provided in any of the sources consulted. It appears that little of the research on blindness in the developing world is population-based [43]. We found a single source that provided population-based rate of blindness as a complication for measles in Africa [44]. Despite the geographical and social difference with Pakistan we included it in the balance sheet as our only point of comparison.

### Adverse effects among the vaccinated

Some adverse effects of measles vaccination did not appear in any of the sources as complications among the unvaccinated with measles. Most of these are local and/or mild reactions (e.g. fever, rash, redness, swelling, etc.), but some, such as anaphylaxis, though rare, do appear to occur among the vaccinated more than among naturally occurring measles cases. Another rare adverse effect is idiopathic thrombocytopenic purpura (ITP). We did not include it in the balance sheet because it seems to be associated with MMR vaccine rather than monovalent measles vaccines used in Pakistan [33]. Other reported adverse effects, lacking evidence of causal association, include Reye's syndrome, oculomotor palsy, optic neuritis, retinopathy, hearing loss, cerebellar ataxia, arthralgia, arthritis, soft tissue reactions, and Guillain-Barre syndrome. The sources consulted also reported certain "described" complications, but without rates of occurrence. These include thrombocytopenia, laryngotracheo-bronchitis, stomatitis, hepatitis, appendicitis and ileocolitis, pericarditis and myocarditis, glomerulonephritis, hyopcalcemia and Stevens-Johnson syndrome [44,45].

The articles from the Cochrane review, our point of departure, did not report rates of death among the vaccinated (i.e. as an adverse effect). We used two cost-benefit articles that provided estimates for this group derived from their models [42,46].

## Results

### The original balance sheet

The summary balance sheet presented in Table 1 reproduces the summary worksheet of the spreadsheet workbook. [The workbook itself is available from the authors.] The bold-font rows of Tables 1 and 2 show the conditions for which the data are most applicable to the South Asia region or, at least, developing country conditions - diarrhoea, pneumonia, blindness and death.

The risk difference and its inverse (NNT) indicate the broad level of public health importance of measles vaccination, taking into account the adverse effects. In the case of anaphylaxis, there is a balance unfavourable to vaccination of about one in a million. Otherwise the balance favouring vaccination is clear: 277 cases of diarrhoea, 61 of pneumonia, 27 of blindness and 14 deaths prevented for every thousand children vaccinated.

### After the baseline survey

Table 1 was based on a set of assumptions that did not prove to be valid in the case of Lasbela as revealed in the baseline survey [47].

Our concern at the design stage that fear of potential adverse effects of vaccination would be an obstacle to vaccination coverage proved to be unfounded. In the baseline survey, household decision makers were asked whether they had heard about bad effects of vaccination, whether they had seen bad effects of vaccination and whether they knew some of the dangerous or severe complications of measles. The answers to these questions, whether positive or negative, had no significant effect on the decision to have the child vaccinated or not.

Only 4% (118/3251) of decision makers had heard of any adverse effect of vaccination. Most of the adverse effects they mentioned were recognised ones, including fever and swelling or pain at the injection site. A few (less than 1%) mentioned things that are not recognised adverse effects of vaccination and among these only a handful mentioned "family planning" signifying the belief that vaccinations will make children sterile or cause them to have only female children in the future. Some 58% (1913/3299) of decision makers reported they knew about some dangerous or severe complications if measles were to get worse. Participants in many focus groups made it clear they knew how serious an illness like measles could be and they mentioned some of the potential consequences of measles, including death.

The baseline survey results also called into question three assumptions on which the data of our original balance sheet were based:

1. Unvaccinated children do not all get measles. The baseline survey found that only 36% of unvaccinated children under five years of age had contracted measles before their fifth birthday. Children from urban households - only about a quarter of the total sample - were more likely to have had measles than rural children (weighted OR 1.64 95%CI 1.36-1.97). When asked how common they thought measles was in their area most decision makers (91%; 3010/3314) said they thought it was rare.

2. Measles vaccination coverage in Lasbela was higher than the rate for Balochistan that we used in the original balance sheet. The PRI, estimated at 76% in our original balance sheet was only 49% according to the baseline survey.

3. The baseline survey revealed that the VER was 41.5%, less than half that assumed in the original balance sheet. The VER calculation is based on all children from 10 to 60 months of age at the time of the survey after excluding those who had measles before the age of 10 months, and children who were vaccinated only after having measles or within one month before having measles. The measles attack rate among vaccinated children (AR_v_) in this group was 12.8% (255/1988) while the attack rate among the unvaccinated (Ar_u_) was 21.9% (386/1759) [47]. We then derived the VER from the formula: VER = 1 - (Ar_v_/AR_u_) [48].

Given these three realities, our original balance sheet considerably overstated the protection that vaccination would provide against the various conditions listed there, as can be seen from Table 2.

The proportions presented in the third column of Table 2 are based on the same risk differences as in Table 1, but the PRI is 0.49 instead of 0.76 and the VER is 0.415 instead of 0.90. Also, instead of assuming that virtually all unvaccinated children would contract the disease, we further adjusted the resulting gains to the proportion of unvaccinated children less than five years of age who ever had measles in Lasbela according to the baseline survey (0.361).

### The real concerns of the communities

Lasbela parents, by and large, were convinced their children should be vaccinated. The baseline showed that 90% of those who make the decisions about vaccination within Lasbela families thought it was worthwhile, 8% did not know whether it was worthwhile or not and only 2% thought it was not worthwhile.

When decision makers were asked about the difficulties households may face in getting their children immunized, more than half reported that access was a problem either because there were no nearby facilities offering vaccination or vaccination teams did not visit (35%), or because of transport problems or poor roads (24%). When asked what would ensure that every child in the household was immunized nearly all responses were related to improving access (92%).

In the end, the balance sheet was not used as part of the Lasbela intervention. Instead, community discussions focused on the relative costs of treating measles cases versus the costs of preventing measles through immunization, as well as on the barriers to obtaining immunization services [3].

## Discussion

### Usefulness of the balance sheet

The balance sheet was not used as part of the intervention because possible adverse effects of vaccination were not a community concern. The major obstacle to improved vaccination rates in Lasbela proved to be the lack of access.

We had anticipated greater concern over the potential adverse effects of measles vaccine than we encountered among Lasbela families. Both the baseline survey and discussions held with non-sample communities during the design phase of the intervention indicated clearly that belief in the efficacy of the vaccine was not a significant factor in household decisions; the overwhelming concern was about the costs of having children vaccinated. These were costs in time, transport and money, resulting in a tendency to postpone having one's child vaccinated and/or discount the likelihood that one's own child might fall prey to the disease [3].

Our theoretical position concerning the rationality of household decisions was too broad to be applicable in the concrete circumstances of Lasbela in 2005-2007. The household decision-making revealed in this experience corresponded more to Herbert Simon's concept of "bounded rationality" [49]. In the words of a contemporary heir to Simon's insights, "...the human mind makes many decisions by drawing on an adaptive toolbox of simple heuristics, not because it is forced to by cognitive constraints, but rather because these fast and information-frugal heuristics are well matched to the challenges of the ... environment"[50]. The environmental challenges most operative in the Lasbela situation were distance and poverty. Knowledge about vaccination was high, attitudes toward it were positive and there was a good deal of discussion within the household about vaccination, but there was little concern about possible adverse effects [2].

Still, people have a right to know about possible adverse effects of any vaccination. We believe that the relevant information should be available to those who request it and in a form that enables them to weigh costs and benefits - in terms of adverse effects and complications - of both being vaccinated and *not *being vaccinated. In other places and under other conditions the potential adverse effects of measles vaccine could be an important deterrent to vaccination. The balance sheet can provide the necessary information in a concise and useful form that will enable people and communities to make rational choices in this regard.

### Vaccine failure

The objective of the Lasbela trial was to demonstrate that informed discussion of costs and benefits could improve demand for vaccination, without relying on improvements in health service delivery. An unvaccinated child still had twice the risk of contracting measles compared to one who was vaccinated. The Lasbela population evidently perceived this as a positive effect, but the odds in favour of vaccination could and should have been much higher.

The low vaccine efficacy indicates that some improvement in vaccine delivery is necessary. The failure rate of over 50% means a partial breakdown in the cold chain or, more likely, its inappropriate management at the point of delivery (for example, partially used multi-shot vials left open or in the light).

### Measles among the unvaccinated

The relatively low rate of measles among the unvaccinated can be explained by the scattered and relatively isolated nature of communities in many rural areas of Lasbela. This apparently creates an environment less conducive to the spread of naturally occurring measles. This conjecture is reinforced by the somewhat higher rates among the minority of children in the denser urban areas [2]. Nine out of ten respondents thought that measles was a relatively rare occurrence in Lasbela and this, too, is likely to have influenced the household cost-benefit calculations about vaccination.

### Limitations of the balance sheet

We were unable to find published data about complications among the unvaccinated or adverse effects of measles vaccination from Pakistan or anywhere in the South Asia sub-region. The extrapolations to South Asia for diarrhoea and pneumonia rates among the unvaccinated are crude estimates at best, based on ratios of diarrhoea and pneumonia occurrence for any cause between one large WHO region and another. Complication rates among the unvaccinated for otitis media, anaphylaxis, convulsions, encephalitis and SSPE reported are based only on developed country conditions, which are quite different.

The balance sheet does not allow for differences in the severity and duration of the conditions it lists. Measles vaccination is known to have a limiting effect in many cases on the severity and duration of common illnesses such as diarrhoea and respiratory disease, but such effects are not taken into account by this instrument. The rates recorded in columns 2 and 3 of Table 1 are for people of any age whereas our gains calculations apply only to children under five years of age. As the practice of generating balance sheets grows, these deficiencies could be improved upon.

We did not determine whether the adverse effects reported by our developed-country sources were reported before or after the introduction of a two-dose regimen for measles vaccine. The likelihood of at least some adverse effects should be higher in the developed countries where two doses is the norm than in developing countries like Pakistan where it is not.

We made no adjustments for vaccine strain. The studies we used for adverse effects came from a review of MMR vaccine. Adverse effects of single measles vaccine cannot always be easily isolated from those of the triple vaccine as reported in our sources. We did not weight the data from the different studies on adverse effects according to their risk of bias.

## Conclusion

The balance sheet may be useful as a communication tool in many other circumstances, but it needs to be tested against up-to-date local evidence in other countries.

If the necessary resources become available, CIET hopes to develop and expand the balance sheet into a web-based tool accessible for critical peer review and eventual public use. We will first have to find ways to overcome as many of the limitations mentioned above as possible. Such a tool could be regularly updated with new research and become a timesaving reference for project managers and heath officials concerned with vaccination.

Figure 1 outlines a possible set of steps toward this end. Since most of the available data on vaccine adverse effects come from developed country sources, the logical starting point would be to post a developed-countries balance sheet and refine it based on critical comments and additional information. The long-term goal, however, should be to have a set of balance sheets for each developing country based on population surveys of measles occurrence. As an intermediary step balance sheets can be developed for WHO regions and sub-regions using data similar to those we used from the WHO South Asia subregion in which Pakistan is located. Country surveys that include information about vaccine adverse effects will help gradually to improve the quality of these regional and sub-regional balance sheets as well.

Country surveys are also helpful for testing vaccine efficacy which, as we have seen, is crucial not only for the balance sheet but for the credibility of the entire vaccination enterprise.

## List of abbreviations used

KT: Knowledge Translation; RD: Risk difference; NNT: Number needed to treat; NNH: Number needed to harm; PRI: Percentage requiring intervention; MMR: Measles, mumps and rubella; WHO: World Health Organization; EMRO: Eastern Mediterranean Region; VER: Vaccine Efficacy rate.

## Competing interests

The authors declare that they have no competing interests.

## Authors' contributions

JF researched the original balance sheet and drafted the methods section. NA designed the balance sheet and contributed to drafting the report. RJL drafted the report.
